# HIV pre-exposure prophylaxis and incidence of sexually transmitted infections in Brazil, 2018 to 2022: An ecological study of PrEP administration, syphilis, and socioeconomic indicators

**DOI:** 10.1371/journal.pntd.0011548

**Published:** 2023-08-11

**Authors:** Paula Knoch Mendonça Gil, Danilo dos Santos Conrado, Ana Isabel do Nascimento, Micael Viana de Azevedo, João Cesar Pereira da Cunha, Gabriel Serrano Ramires Koch, Camila Guadeluppe Maciel, Alisson André Ribeiro, Antonio Conceição Paranhos Filho, Márcio José de Medeiros, Cláudia Du Bocage Santos-Pinto, Everton Falcão de Oliveira

**Affiliations:** 1 Programa de Pós-Graduação em Doenças Infecciosas e Parasitárias, Universidade Federal de Mato Grosso do Sul, Campo Grande, Mato Grosso do Sul, Brasil; 2 Faculdade de Medicina, Universidade Federal de Mato Grosso do Sul, Campo Grande, Mato Grosso do Sul, Brasil; 3 Instituto Integrado de Saúde, Universidade Federal de Mato Grosso do Sul, Campo Grande, Mato Grosso do Sul, Brasil; 4 Faculdade de Engenharias, Arquitetura e Urbanismo e Geografia, Universidade Federal de Mato Grosso do Sul, Campo Grande, Mato Grosso do Sul, Brasil; 5 Instituto Politécnico, Universidade Federal do Rio de Janeiro, Macaé, Rio de Janerio, Brasil; Tulane University School of Public Health and Tropical Medicine, UNITED STATES

## Abstract

**Background:**

Human immunodeficiency virus (HIV) pre-exposure prophylaxis (PrEP) is one of the pillars of a combination prevention strategy for reducing the risk of new infections caused by HIV. The daily use of antiretroviral drugs by individuals who are not infected with HIV is required to prevent infection. Although its efficacy has been well established in the literature, in recent years, the decreased supply of antiretroviral drugs has been associated with an increase in the incidence of sexually transmitted infections (STI) and changes in the social determinants of health. An ecological study was conducted covering a five-year period (2018–2022), starting from the year of initiation of PrEP administration in Brazilian state capitals.

**Principal findings:**

Descriptive analysis was performed, and the spatial distribution of study data was taken into account. Correlation analysis was used to assess the association between PrEP administration, the incidence and detection rate of STI, and socioeconomic data. The southern region showed the highest incidence rates of STI, but the northern and northeastern regions demonstrated the worst socioeconomic indicators, especially those related to illiteracy and basic sanitation. PrEP administration was significantly correlated with illiteracy (ρ = -0.658), per capita income (ρ = 0.622), public garbage collection (ρ = 0.612), syphilis (ρ = 0.628) and viral hepatitis (ρ = 0.419) incidences. Further, all STI were significantly associated with illiteracy and per capita income.

**Significance:**

Our findings highlight the need to continue exploring PrEP use and rising syphilis rates. In terms of policy, PrEP administration appears to be inversely associated with regions of greater social vulnerability. Further efforts should focus on the social determinants and health needs of this population to improve access to PrEP and reduce social disparities.

## Author summary

The human immunodeficiency virus (HIV) infection, if left untreated, can lead to acquired immunodeficiency syndrome, known as AIDS. It is a global epidemic infectious disease that remains incurable but has treatment and prevention strategies. Several studies have been conducted to identify new technologies to control and cure this disease, such as vaccines and new drugs. Furthermore, there are several strategies for combined prevention of this infection, including HIV pre-exposure prophylaxis (PrEP), which has been adopted as a Brazilian public health policy and is indicated for individuals who are not infected with HIV. Despite being effective, it only prevents HIV infection and not other sexually transmitted infections (STI). It is estimated that socioeconomic indicators may be correlated with and directly impact access to prophylaxis. In light of this scenario, an ecological study was conducted to verify whether PrEP administration is associated with an increase in STI, and whether its access and coverage are associated with the socioeconomic conditions of the population. It was observed that PrEP administration and the most vulnerable socioeconomic indicators showed an inversely proportional association; these results could guide actions and investments focused on public health, aiming to reduce social disparities and inequalities in access to this strategy.

## Introduction

Human immunodeficiency virus (HIV) pre-exposure prophylaxis (PrEP) is one of the pillars of a combined prevention strategy for reducing the risk of new HIV infections [1]. The daily use of antiretroviral drugs by individuals not infected with HIV is required to prevent infection in at-risk populations [2]. In 2017, the combination of tenofovir and emtricitabine (TDF/FTC 300/200 mg) was incorporated into the Sistema Único de Saúde (SUS), Brazil’s unified health system [3], for populations at risk for HIV infection who are recommended to be on antiretroviral drugs for PrEP.

Initial studies, such as IPrEX [4], IPERGAY [5], and PROUD [6], demonstrated the effectiveness of PrEP as an HIV prevention strategy, with up to an 86% reduction in infections. The PrEP Brazil [7] and Combina [8] studies contributed to the implementation of the strategy in the SUS [1], which was indicated for four main priority groups: men who have sex with men (MSM) with a history of sexually transmitted infections (STI) in the last six months, commercial sex workers (CSW), transgender people, and HIV serodiscordant couples, until August 2022 [9]. In 2022, a new version of the Clinical Protocol and Therapeutic Guidelines was published, which expanded the eligibility criteria for PrEP use [10], recommending prophylaxis to all people at an increased risk of acquiring HIV infection, regardless of sexual orientation or practice.

Currently, there are two regimens for oral PrEP use: continuous, which consists of routine daily use of the medication, and PrEP on demand, in which the use occurs before and after exposure or sex without the use of a condom [10]. Both regimens reduce the risk of HIV infection by more than 85% [5,6,11]. Injectable PrEP with cabotegravir is being tested, and preliminary data have shown a 66% to 89% reduction in the risk of infection [12,13].

One of the concerns with PrEP use is the rising number of cases of other STI [14]. This phenomenon of a causal relationship between PrEP use and an increase in other STI is still not a consensus; however, it reinforces the importance of combination prevention strategies [15].

Despite its effectiveness in reducing the risk of HIV transmission, the supply and population coverage of PrEP may be affected by social determinants of health [16], such as socioeconomic and demographic factors that negatively impact access to health services [17,18]. As PrEP was initially prioritized for vulnerable populations, considered to be at higher risk for STI, and who historically suffer from marginalization and stigma, these factors may represent structural barriers to accessing PrEP, reducing access and adherence and, consequently, thwarting the main objective of this intervention to reduce the risk of infection [19].

Based on this hypothesis, we aimed to explore whether PrEP administration is associated with an increase in STI and whether access and coverage are associated with the socioeconomic conditions in the 27 Brazilian state capitals (26 state capitals and the Federal District, Brasilia).

To date, this study is one of the first assessments of PrEP administration using national coverage data in Brazil, and we analyzed its spatial distribution and its association with socioeconomic data. The results are useful to show the possible effect of social disparities on access to PrEP and on the risk of PrEP use for other STI. These results are particularly useful for Brazil, which was a world reference in public treatment and control policies for HIV/AIDS and served as a model for other health systems [20, 21]. In this sense, the results presented can contribute toward program directions to improve access in more vulnerable areas of the country.

## Methods

### Ethics statement

This study was based on secondary data from the public domain available in the official information systems of the Ministry of Health and IBGE. Therefore, ethical review was not required.

### Study design, setting, and period

This is an ecological study with the 26 Brazilian state capitals and the Federal District as the units of analysis. The study assessed the association between PrEP administration, the incidence of compulsory STI notification (HIV, acquired/gestational syphilis, and viral hepatitis) data from 2018 to 2022, and socioeconomic and sanitation status from the 2010 decennial census, which are updated periodically with projections for the state capital areas.

### Study data

The STI included in this study were those routinely analyzed in Brazil, as they were on the national list of compulsory notifiable diseases throughout the country. The health data reported STI for all ages and genders in the covered population from 2018 to 2022, in parallel to the Brazilian Institute of Geography and Statistics (IBGE) data, which reported that for the entire population.

The PrEP administration data included individuals who had at least 1 drug refill preceded by HIV testing in each year assessed during the study period. According to the Brazilian protocol for the administration of PrEP in force between 2018 and August 2022, only people over 18 years of age could obtain medication from the SUS [3,9]. Therefore, the PrEP administration indicator calculated in this study considered only the population over 18 years of age.

Socioeconomic and sanitation data include those that were routinely studied as social determinants of health and that were available for the study unit analysis.

- All study data were obtained from public domain nationwide databases as follows:
- Monthly PrEP administration data, which refers to PrEP users with at least 1 drug refill preceded by a HIV testing in each year assessed during the study period: https://www.gov.br/aids/pt-br/assuntos/prevencao-combinada/prep-profilaxia-pre-exposicao/painel-prep
- HIV/AIDS cases in all ages and genders: https://indicadores.aids.gov.br/
- Viral hepatitis cases in all ages and genders: http://indicadoreshepatites.aids.gov.br/. Since data were not available for 2022, data from 2018–2021 were used.
- Syphilis cases in all ages and genders: http://indicadoressifilis.aids.gov.br/
- Socioeconomic data, which include illiteracy rate, Gini coefficient, per capita income, the proportion of residents with regular garbage collection services, the proportion of residents with sanitary facilities, and water supply: https://censo2010.ibge.gov.br/sinopse/
- Population estimates for Brazilian state capitals from 2018 to 2022: https://www.ibge.gov.br/estatisticas/sociais/populacao/9103-estimativas-de-populacao

Health and IBGE data reports overlapped in the entire metropolitan region, which allowed for data comparability.

### Statistical analysis

Tables and frequency distribution charts were used for the characterization of the study data.

Cumulative incidence was used as a measure of disease frequency for HIV/AIDS and viral hepatitis, whereas the detection rate was used for syphilis. The estimated population for each year was used as the denominator for both measures. The number of PrEP users in the year was divided by the estimated population aged 18 and over of each unit analysis to obtain the proportional PrEP administration. For the cumulative measures from 2018–2022, the mid-period population was used as the denominator.

**Fig 1:**
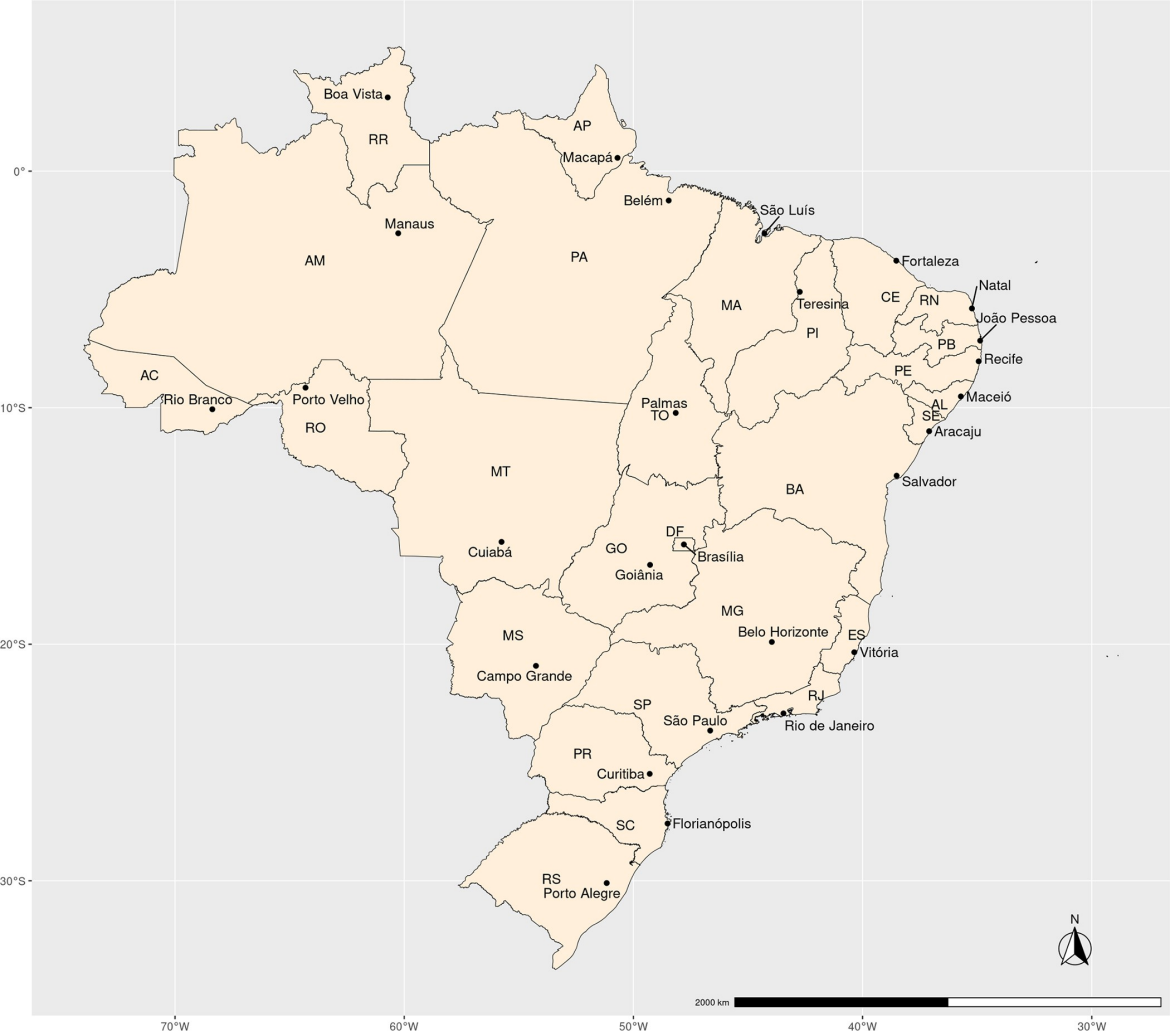
Study area. Map was made in R version 4.1.2 (package *ggplot2*) using data from IBGE.

Choropleth maps were used to represent the spatial distribution of the data analyzed in this study. Although the data referred to the capital cities, the geographical limits of the Brazilian states were used for geographical representation. Dots have been included to indicate state capitals (Fig 1). The basemap shapefile onto which the data has been plotted is in the public domain and was extracted from IBGE: https://www.ibge.gov.br/geociencias/downloads-geociencias.html. Maps were produced in R version 4.1.2 (https://www.r-project.org/) and packages *ggplot2* and *sf* were used.

Spearman’s rank correlation coefficient was used to assess the association between PrEP administration, the incidence and detection rate of STI, and socioeconomic/sanitation data. To describe the general pattern between PrEP administration and other variables, locally weighted scatterplot smoothing (LOWESS) was used [22–24]. The software R version 4.1.2 (https://www.r-project.org/) was used for the analyses, and a significance level of 5% (α = 0.05) was adopted. Packages *ggplot2*, *tidyverse* and, *descr* were used.

## Results

PrEP administration in Brazil started in 2018, and by the end of that same year, 23 Brazilian state capitals and the Federal District were administering it. The last state capitals to begin operations were Teresina (PI) and Maceió (AL) in 2019 and Rio Branco (AC) in 2020.

**Fig 2:**
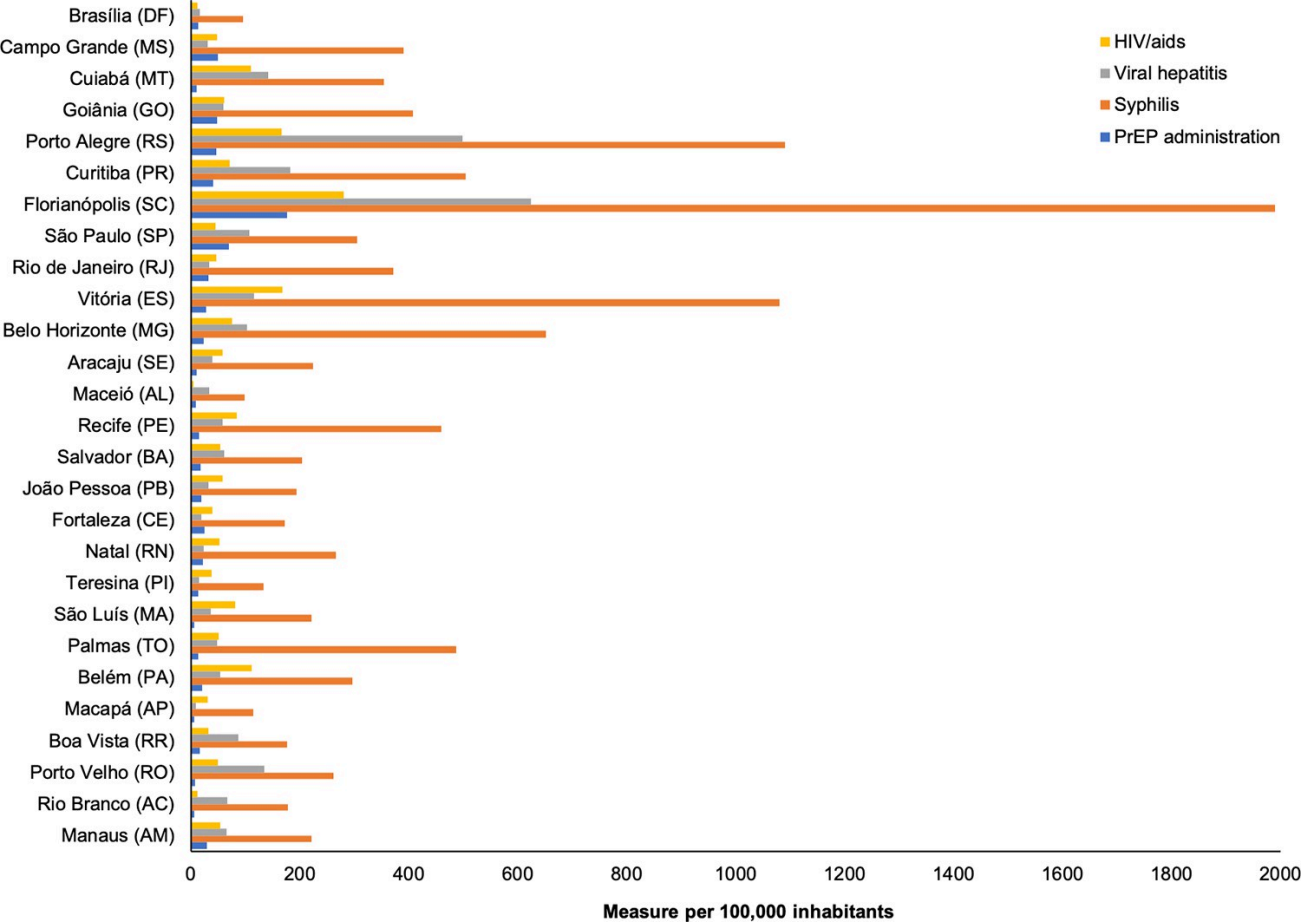
PrEP administration and cumulative STI incidence per 100,000 population in Brazilian capital states, 2018–2022.

São Paulo (SP), Rio de Janeiro (RJ), and Curitiba (PR) had the highest PrEP administration values, while Rio Branco (AC) and Macapá (AP) had the lowest. When considering the proportional administration per 100,000 inhabitants, Florianópolis (SC), São Paulo (SP), and Vitória (ES) stood out with the highest values, and Maceió (AL), Rio Branco (AC), and Macapá (AP) had the lowest (Fig 2 and S1 Table).

For the STI cases, it was noted that Maceió (AL) and the Federal District had the lowest incidence. The cites of Maceió (AL), Rio Branco (AC), Macapá (AP), São Luís (MA), Federal District, Recife (PE), and Teresina (PI) had the lowest proportion of STI cases. Nevertheless, the capitals with the highest cumulative incidence of STI (Florianópolis [SC], Vitória [ES], and Porto Alegre [RS]) also had the highest proportional PrEP administration. A case that showed a different profile from those previously mentioned was São Paulo (SP), where high proportional PrEP administration was not accompanied by a higher incidence of STI (Fig 2 and S1 Table). Even with the increase in the administration of PrEP in São Paulo, the occurrence of STI, especially syphilis, was similarly to that of the other capitals, unlike Florianopolis, where there was a huge increase in the syphilis detection rate, which differed from the other capitals.

**Fig 3:**
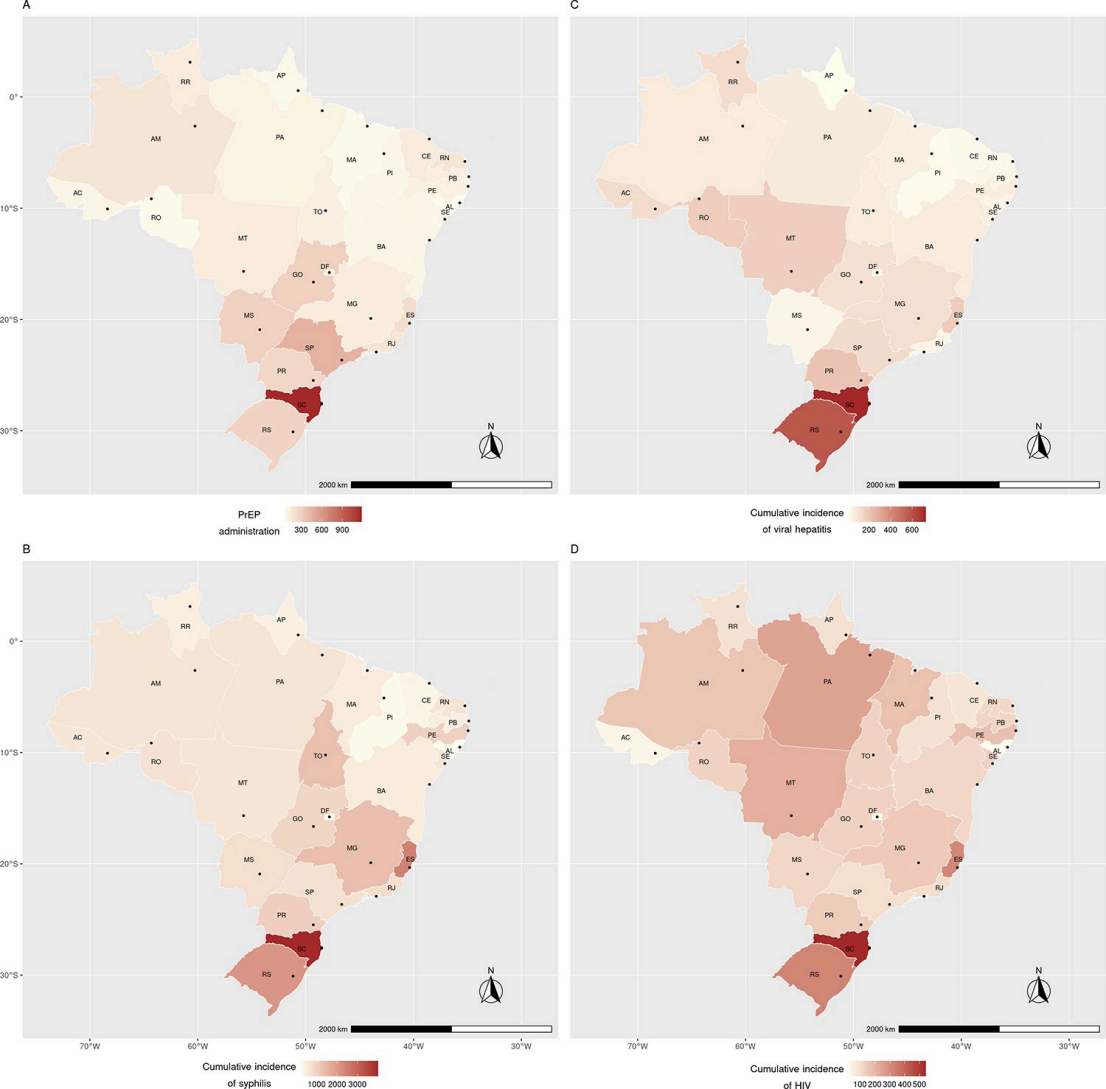
Spatial distribution of PrEP administration and incidence of sexually transmitted infections in Brazilian capital states, 2018–2022. Map was made in R version 4.1.2 (packages *ggplot2* and *sf*) using data from IBGE.

Fig 3 illustrates the spatial distribution of proportional PrEP administration and measures of cumulative new STI cases for the period of 2018–2022, which are detailed in S1 Table. We highlight the highest rate of syphilis detection in capitals in the southern region (Florianópolis [SC], Porto Alegre [RS], and Curitiba [PR]) as well as Vitória (ES), Palmas (TO), Recife (PE), and Minas Gerais (MG). The highest incidence of viral hepatitis was observed in certain capitals in the northern region (Rio Branco [AC] and Boa Vista [RR]), midwestern region (Cuiabá [MT]), southeast region (Vitória [ES]), and all capitals in the southern region (Florianópolis [SC], Porto Alegre [RS], and Curitiba [PR]). Regarding the spatial distribution of HIV/AIDS incidence, capitals São Luís (MA), Belém (PA), Cuiabá (MT), Recife (PE), Vitória (ES), Florianópolis (SC), and Porto Alegre (RS) stand out. A similar pattern was observed between the PrEP distribution and syphilis occurrence in Brazil’s central belt (Fig 3).

Regarding the temporal progress of PrEP administration, the frequency per 100,000 inhabitants in the five evaluated years increased significantly by 13,100% and 9,300% in the capitals of Campo Grande (MS) and Cuiabá (MT), respectively. However, the capitals that had a smaller increase were Manaus (AM) and Porto Alegre (RS), at 29% and 328%, respectively (S2 Table).

**Fig 4:**
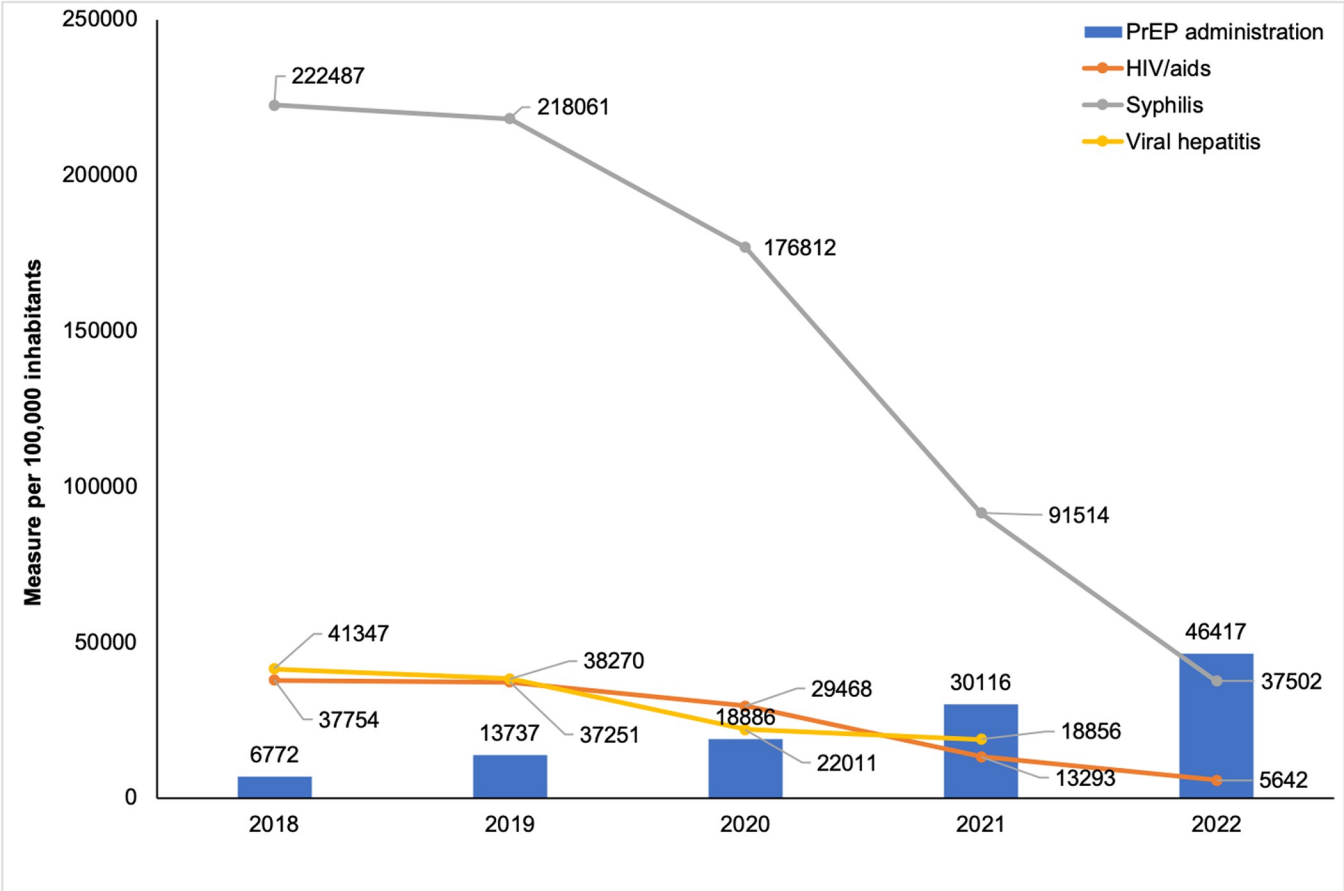
Time progress of PrEP administration and STI incidence in Brazilian capital states, 2018–2022.

By analyzing the occurrence of new cases of STI, a reduction in the incidence/detection rate was observed when comparing the first and last years of the series evaluated (S2 Table). When the temporal evolution of proportional PrEP administration was evaluated together with STI, considering data from all capitals, it was observed that there was a continuous increase in administration, which was accompanied by a drop in the occurrence of STI, especially syphilis, from 2020 to 2022 (Fig 4).

**Fig 5:**
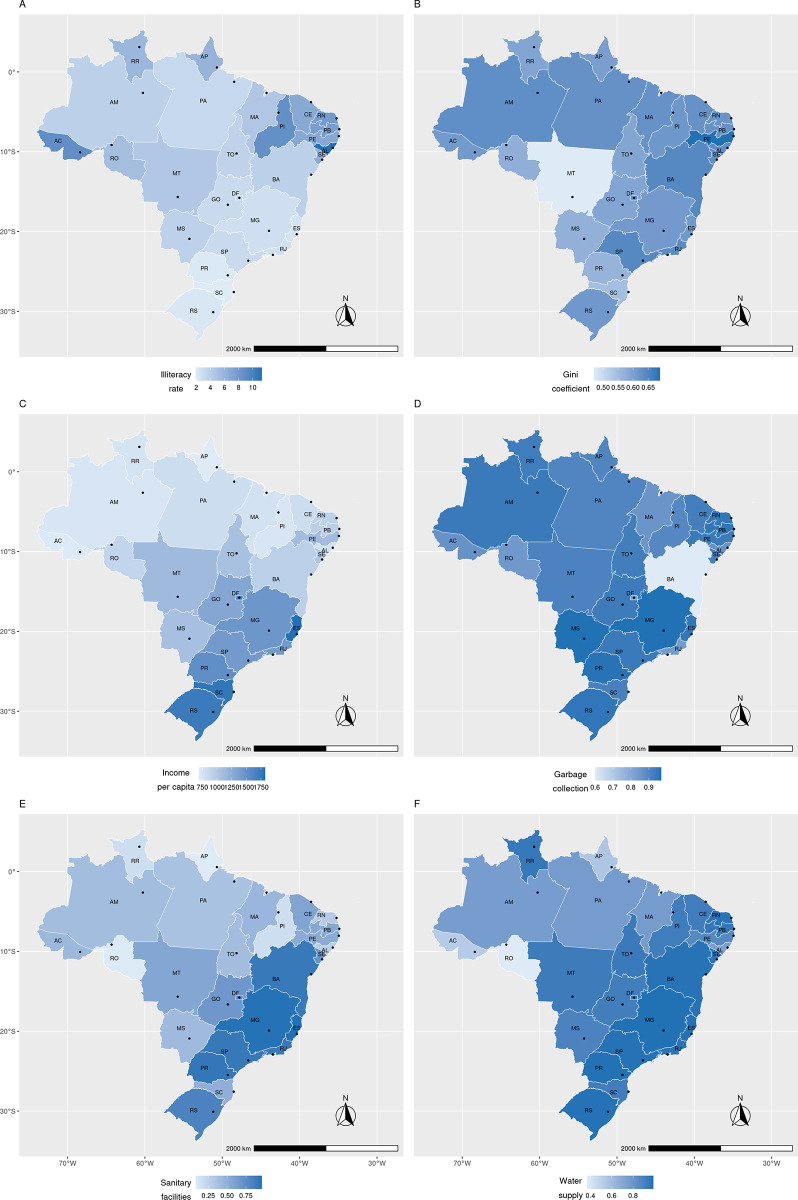
Spatial distribution of socioeconomic indicators in Brazilian capital states in 2010. Map was made in R version 4.1.2 (packages *ggplot2* and *sf*) using data from IBGE.

Fig 5 and S3 Table present the distribution of socioeconomic indicators included in the study. The highest illiteracy rates were concentrated in the northern and northeastern regions, while the lowest rates were in the southern and southeastern regions. In relation to the Gini coefficient, capitals with the least social inequality were found in the midwestern, southern, and northern regions. As for per capita income, the highest values were observed in the southeastern and southern regions. Some capitals in the five regions stood out for presenting the highest percentages of coverage, such as Natal (RN), João Pessoa (PB), Belo Horizonte (MG), Vitória (ES), Campo Grande (MS), Curitiba (PR), and Porto Alegre (RS). For sanitary facilities and water supply, states located predominantly on the west coast of the country (southern and southeastern regions) showed the best results (Fig 5).

**Fig 6:**
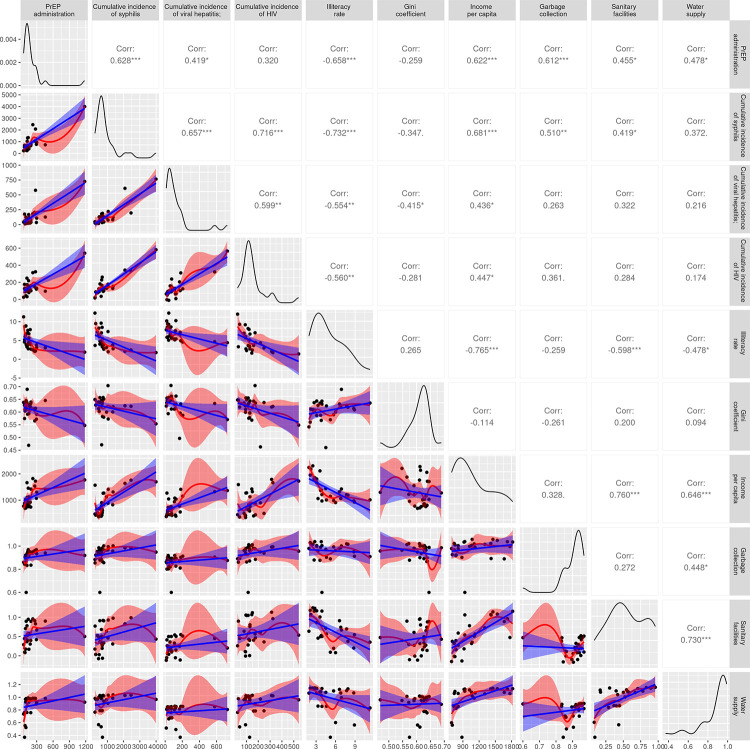
Scatter plots and matrix correlations of study data. ***p<0.001; **p<0.05; *p = 0.10.

Fig 6 presents the correlation matrix and dispersion diagrams of association analyses. Notably, the proportion of PrEP administration showed an inverse and significant correlation with the incidence of all STI, the inverse correlation with the occurrence of syphilis being the largest among the three STI analyzed. PrEP administration showed a significant association with all socioeconomic indicators except for the Gini coefficient. It can be highlighted by the negative (inversely proportional) and the positive (directly proportional) correlations of PrEP administration with the illiteracy rate and income per capita, respectively.

**Fig 7:**
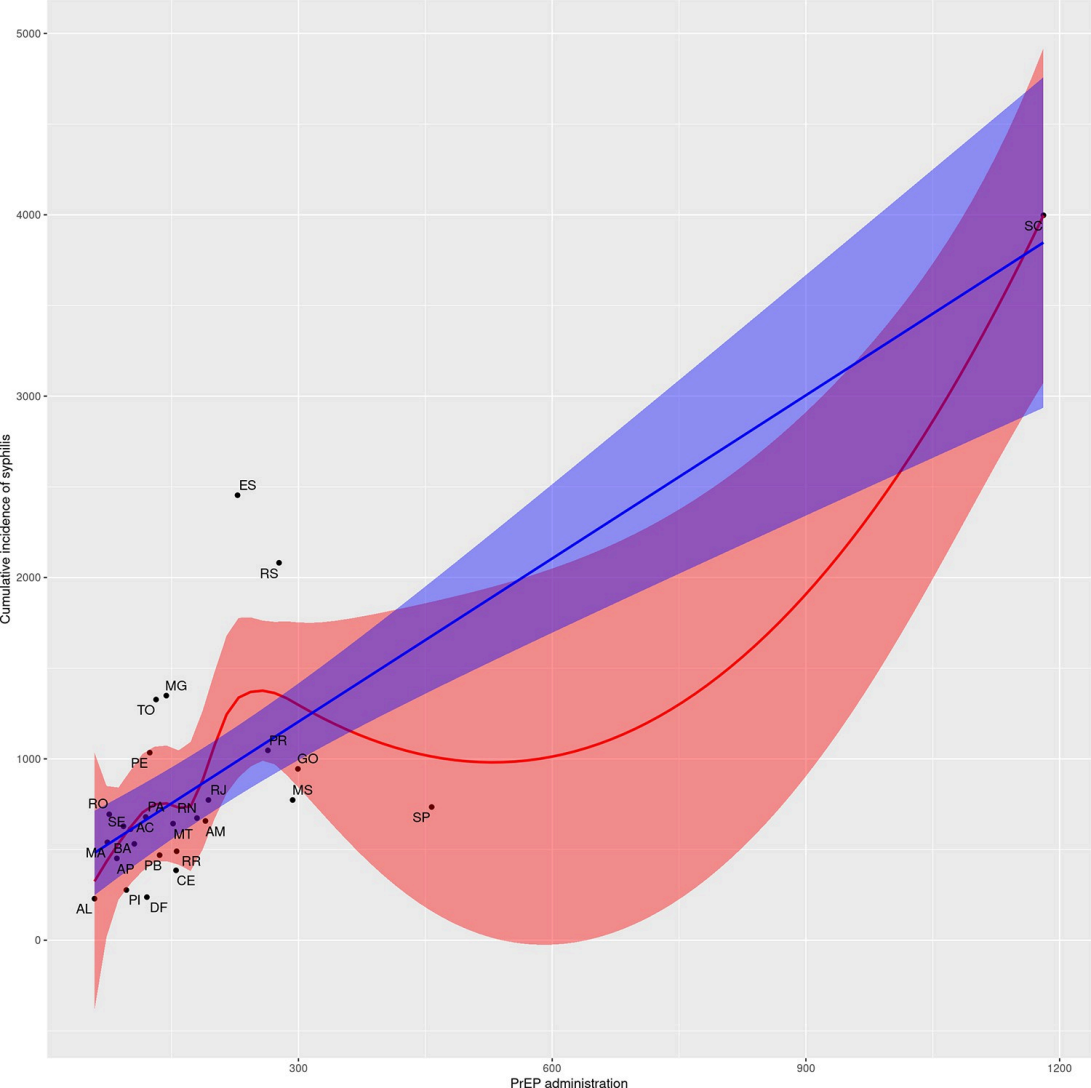
Scatter plots for PrEP administration and syphilis detection rate.

**Fig 8:**
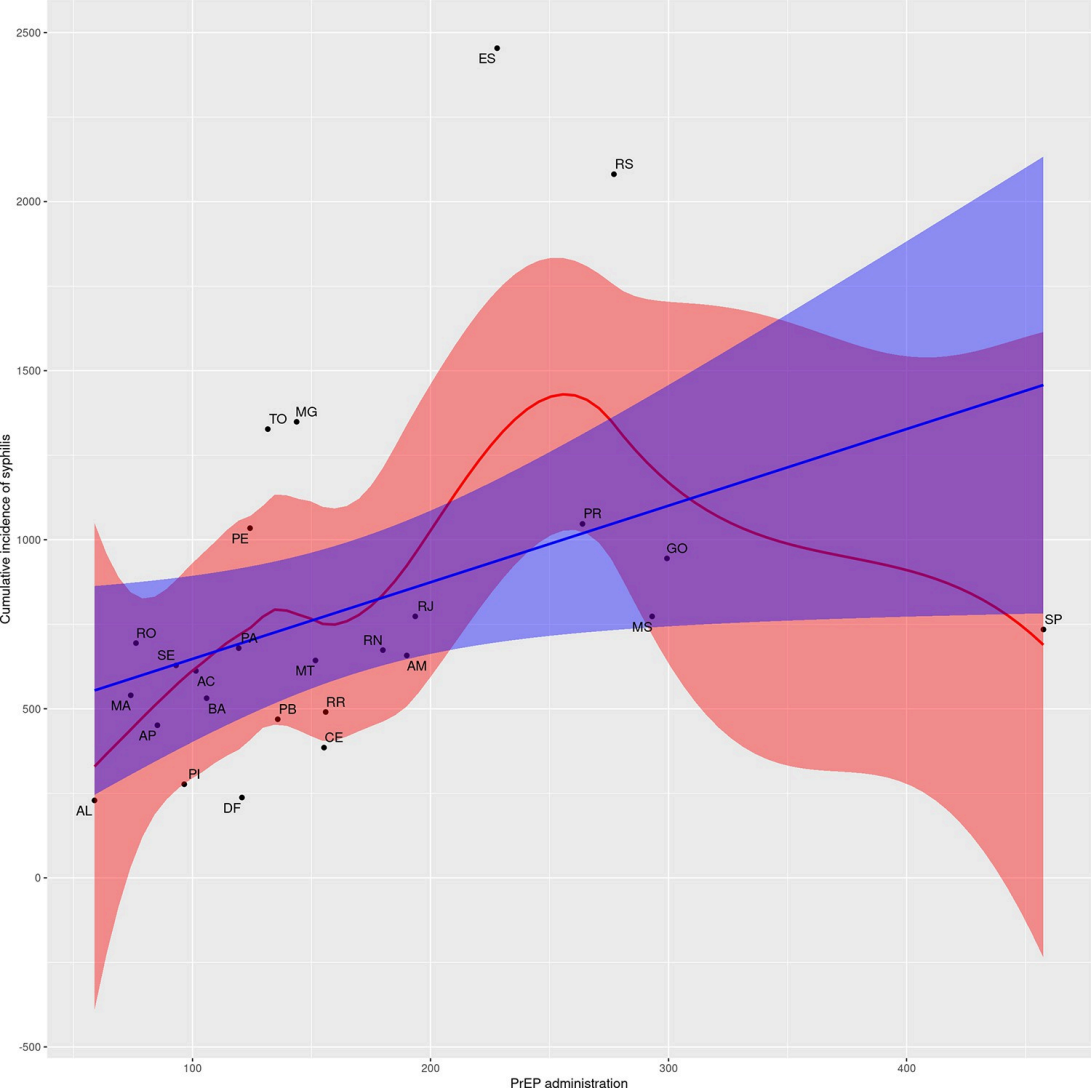
Scatter plots for PrEP administration and syphilis detection rate (without Florianopólis).

Data from Florianópolis differed from that of other capitals, characterizing it as an outlier. For comparison purposes, a new correlation and dispersion matrix without Florianópolis was constructed (S1 Fig). To illustrate the different behavior of Florianópolis, Figs 7 and 8 present the general pattern between the variables of PrEP administration and syphilis detection rate with and without this student capital, respectively. Although the associations related to the PrEP and syphilis data remain significant when Florianopolis is removed from the analysis, the magnitude of the correlation coefficients reduces slightly.

Regarding HIV/AIDS incidence, there was a negative correlation with education and a positive correlation with per capita income, garbage collection, and sanitary facilities, and no association with the Gini coefficient or water supply.

Regarding the syphilis detection rate, there was a correlation with all socioeconomic indicators, being inversely proportional to education and the Gini coefficient and directly proportional to garbage collection, sanitary facilities, and water supply.

The incidence of viral hepatitis showed a positive correlation with per capita income and sanitary facilities and a negative correlation with education and the Gini coefficient. There was no significant association between the incidence of viral hepatitis and the indicators related to garbage collection and water supply.

## Discussion

We analyzed the first five years of PrEP administration data in Brazil, revealing important findings in the correlation and spatial distribution of health data related to the use of prophylactic therapy, compulsory notification of STI, and socioeconomic differences among the regions, and particularly among the northern and northeastern Brazilian capitals.

In Brazil, considering only state capitals, which are also the most densely populated cities of Brazil, PrEP administration was more concentrated in the southeastern, southern, and midwestern regions, which may be related to a higher population density, a higher percentage of STI testing, and consequently, a higher number of people eligible to use PrEP [9]. In addition, the existence of specialized reference services for STI in these cities can be considered, which are units trained to address the needs of target populations, including PrEP supply [16].

Although dispensing occurs in all Brazilian states, barriers to PrEP access are believed to exist, especially for more vulnerable population groups, such as transgender and diverse gender individuals, who often lack access to medical affirmation of their gender or essential sexual health services, in addition to the stigma experienced by these populations [20, 1]. Other limiting factors include PrEP adoption, cost, frequency of STI counseling and treatment services, possible drug interactions, and labeling of PrEP users as HIV positive [25]. PrEP may be offered at no cost, but users still incur indirect costs such as those associated with travel/transport and lost wages from long waiting hours in most facilities [26–29]. A further limitation is the slow rollout of PrEP and special criteria for administration. Additionally, regular HIV testing is required, which affects diverse populations differently [3].

Regarding the incidence of syphilis, viral hepatitis and HIV, a higher concentration was observed in the southern region, although some studies suggest that areas with a lower human development index have higher incidence rates of HIV, syphilis, and viral hepatitis [30–35]. Some hypotheses that help explain this scenario might be related to the need for regular STI tests for patients using PrEP, which could lead to a higher detection rate in the population with more access to prophylaxis [10,33]. A study carried out on the epidemiological aspects of syphilis in southern Brazil also reinforces this finding, by showing a significant increase in the occurrence of this STI from 8.37% in March 2012 to 21.83% in March 2017 [34].

Despite some contradictions in the results and findings of the studies, it should be noted that the occurrence of diseases in a population, especially in a country of continental dimensions like Brazil, is influenced by several factors, such as biological, environmental, social, cultural, educational, economic, political, and even geographical issues [36].

Notably, while a rising syphilis epidemic has been identified, it cannot be said that it is necessarily and exclusively a notification problem, nor can the problem be attributed to PrEP. However, some relevant issues can be raised, such as the penicillin shortage crisis, which occurred in Brazil between 2013 and 2017 and which was responsible for a huge increase in the number of syphilis cases in the country over that period [37,38]. After improving the supply of penicillin from 2018 onwards, it was already possible to notice a stabilization in the disease detection rate, with a sharp decline in 2020, possibly related to the underreporting during the COVID-19 pandemic [38]. Also, the changes brought about during the pandemic may have led to a decrease in sexual partners, health service use, and rapid test use [33,39,40]. The same scenario of reduced incidence of STI during the COVID-19 pandemic has also been observed in other countries where PrEP is available [41–44]. In addition to the impact of the COVID-19 pandemic, several gaps remain that could potentially explain the rapid and significant decline in the detection rate of syphilis in Brazilian capitals.

The HIV/AIDS epidemic in Brazil has a prevalence of approximately 0.6% in the general population, and its geographic variation has shown a decrease in the southeast and an increase in the northern and northeastern regions [35], which is in line with data from our study.

Our results showed that higher PrEP administration was accompanied by an increase in the incidence of STI in some state capitals, especially in the southern region. However, this was not the case for all the capitals, as observed in Fortaleza and São Paulo, the two state capitals with the highest population density in Brazil. Some studies have described an increased STI incidence in PrEP users compared to those not using this prophylactic method [29]. In the IPREX study [4], the frequency of syphilis at baseline was approximately 14% of study participants, with an incidence of 7.3 cases per 100 persons/year over time. According to Volk et al. [45], STI incidence after 6 months of PrEP use was 30%, of which 3.3% was syphilis-related; after 12 months of PrEP, it was 50%, of which 5.5% was syphilis-related. Another study demonstrated an increase in STI, such as gonorrhea, syphilis, and chlamydia, in regular PrEP users [46]. Other studies showed a positive association between the use of PrEP and the incidence of syphilis [3, 10], which partially corroborate the findings of our study, in which in the analysis of the temporal evolution, there was a decline in the detection rate of syphilis but a positive correlation between the PrEP administration and syphilis occurrence.

In a study carried out in Brazil concerning sexual behavior, a low prevalence of condom use was observed, in addition to important socioeconomic and demographic disparities [47]. Based on this report and our results, it is plausible to speculate that in Brazil, PrEP use was not accompanied by enhanced condom use. This context points the need to rethink, strengthen, and expand public policies in the sexual and reproductive health field, seeking to preventing risky sexual behavior and comprehensively promoting the use of condoms, including double protection.

The United Nations classifies Brazil as one of the most unequal countries in the world. The country has highly developed regions, such as the southern and southeastern regions; simultaneously, it is possible to find others with much lower development rates, especially in the northern and northeastern regions [48]. These differences are driven by social determinants of health related to social, economic, cultural, ethnic/racial, psychological, and behavioral factors that can directly influence risk factors [49], the occurrence of health problems, and exposure to STI [50,51]. These determinants also influence the ability of the health sector to respond and provide high quality services with adequate coverage.

The weight of social determinants of health, especially those included in our study, on STI risk has been described in the literature [16,17,49]. Starting from this premise, our study was developed under the hypothesis that there is also an association between socioeconomic conditions and access to and population coverage of PrEP, which was observed after data analysis. A multicenter study demonstrated that structural barriers, such as poverty, racism, gender inequality, and criminalization of sex work, impact access to health services and negatively influence PrEP access. It also highlighted factors related to health services that can be barriers to vulnerable populations accessing these services as well as PrEP, such as lack of adaptation to patients’ life and work contexts and stigma and discrimination related to sexuality and gender identities [16].

PrEP administration should still follow HIV testing, but HIV testing is often an impediment to care-seeking, especially for special populations such as MSM and CSW who are at the highest risk and may not often attend general population primary care facilities [52,53]. Populations with the greatest concentration of STI, including HIV, are also socially and politically marginalized, and not sufficiently engaged in policy-making and the design and delivery of services. In effect, their right to preventive health services is violated [1].

Considering that STI persist as a global public health problem, with an estimated 376 million new cases per year [54], studies on the evaluation of policies and strategies to control these diseases, as well as on factors related to coverage of PrEP, are essential. Considering published reports in Brazil on the role of education in PrEP use [55–57], the integration between different sectors, such as education and health, is essential for joint action in strategies to expand access to PrEP. Our results indicate that a low educational level can act as a barrier to accessing PrEP.

This study had some limitations. One limitation was the impossibility of defining the specific clinical and sociodemographic profiles of PrEP users as well as those without access to PrEP; it was also impossible to define other characteristics of these populations, such as age, skin color, gender, and sexual behavior and sexual orientation, and whether these individuals were within a key risk group for HIV. It was also impossible to determine the occurrence of STI among regular PrEP users. Given that this study utilized an ecological study design with routine available data, it was not possible to gather health data on STI, nor were we able to identify people who were at increased risk for STI and who had an indication for the use of PrEP within our reference population (total resident population). For example, syphilis data is very gender patterned, and most cases are identified in women on routine screening during pregnancy, a population much larger than MSM. There were also issues concerning the reporting of the health data: each disease and treatment have particular monitoring histories and are affected differently by secular trends that may influence reporting during the study period. However, if a given disease is increasing or decreasing in a given population, further investigations are necessary to determine its causes and the subpopulations affected. Notably, the data were also limited as the IBGE has a greater than ten-year lag in data on socioeconomic indicators. Moreover, there were major limitations in understanding the relationship among PrEP, condom use, and syphilis. It was not possible to carry out the analysis by sexual orientation, as these data were not included in the Brazilian Notifiable Diseases Information System and in the IBGE.

While many state that Brazil’s AIDS program is a model for the world, that would more properly apply to the program earlier in its life. In 2016, during a political transition of the federal government, the AIDS program was combined into a larger department and has received less attention; funding for non-governmental organizations (NGO) serving MSM finally dried up, targeted communication to MSM about prevention was reduced, and eventually shortages of antiretroviral drugs occurred. HIV seropositivity rates, especially for young MSM, grew quickly during this time, and this has been well documented [58–60]. Finally, the knowledge of PrEP and its purpose was extremely limited between 2018 and 2021 in many areas in Brazil, perhaps also due to limited attention from a central AIDS program or promotion through NGO. Ultimately, the complicated nature of the relationships between PrEP roll-out and administration, condom use, behavioral disinhibition, and STI cannot be teased apart with the data sources and design of this study. The implication of the study, however, is that it is not just a simple relationship of provision and response, but it is moderated by geographic location, socioeconomic status, and other variables. Nevertheless, it is important to understand the limitations of using routine data to answer important hypotheses; it is also important to highlight that these are limitations inherent to ecological studies, where there is no intention of making direct associations but raising hypotheses about the group of observations.

## Conclusion

Based on these results, it is possible to highlight the need for a closer look at PrEP access for groups greatly impacted by social determinants. In addition to investing in actions guided by public policies related to PrEP, a broader focus is needed to reduce existing social disparities in the population. Future analyses that deepen the unequal experience of users and the perspective of healthcare professionals regarding this policy are necessary for the continued strengthening of access to PrEP and other strategies for combined prevention. It is necessary to carry out further research on syphilis and the use of PrEP to elucidate these issues alongside the gaps identified in our study. Finally, the relevance of PrEP to public health is highlighted as a strategy to reduce cases of HIV and, consequently, other STI through surveillance of these users and expansion of coverage of vulnerable populations to infectious diseases.

## Supporting information

S1 Table Distribution of frequencies of PrEP administration and compulsory notification of STI in Brazilian capital states from 2018–2022. (PDF)

S2 Table PrEP administration and STI occurrence in Brazilian capital states from 2018–2022. (PDF)

S3 Table Socioeconomic indicators of Brazilian capital states in 2010. (PDF)

S1 Fig Scatter plots and matrix correlations of study data (without Florianopólis [SC]). ***p<0.001; **p<0.05; *p = 0.10
(TIFF)
