# Supplementary material for: HIV pre-exposure prophylaxis and incidence of sexually transmitted infections in Brazil, 2018 to 2022: An ecological study of PrEP administration, syphilis, and socioeconomic indicators
Source: PLoS Negl Trop Dis. 2023 Aug 11;17(8):e0011548. doi: 10.1371/journal.pntd.0011548 (PMC10446216; doi:10.1371/journal.pntd.0011548)
Supplement: S1 Table — (PDF) [file pntd.0011548.s001.pdf]

Supporting information

HIV pre-exposure prophylaxis and incidence of sexually transmitted infections in Brazil, 2018 to 2022: an ecological study of PrEP administration, syphilis, and socioeconomic indicators

Paula Knoch Mendonça Gil, Danilo dos Santos Conrado, Ana Isabel do Nascimento, Micael Viana de Azevedo, João Cesar Pereira da Cunha, Gabriel Serrano Ramires Koch, Camila Guadeluppe Maciel, Alisson André Ribeiro, Antonio Conceição Paranhos Filho, Márcio José de Medeiros, Cláudia Du Bocage Santos-Pinto, Everton Falcão de Oliveira

S1 Table. Distribution of frequencies of PrEP dispensing and compulsory notification of STI per 1000,000 inhabitants in Brazilian state capitals, Brazil, 2018–2022

Brazilian region	State capital (State)	PrEP administration*	PrEP administrations per 100,000 inhab.	HIV/AIDS	Syphilis	Viral hepatitis
Northern	Manaus (AM)	3960	29	54	222	65
	Rio Branco (AC)	291	7	12	178	67
	Porto Velho (RO)	316	8	50	262	134
	Boa Vista (RR)	537	17	32	177	87

	Macapá (AP)	343	6	31	114	9
	Belém (PA)	1560	21	111	297	54
	Palmas (TO)	343	13	51	487	49
Northeast	São Luís (MA)	673	7	82	222	37
	Teresina (PI)	722	14	39	133	15
	Natal (RN)	1490	22	53	267	24
	Fortaleza (CE)	3832	25	39	172	19
	João Pessoa (PB)	1086	20	59	194	33
	Salvador (BA)	3051	18	55	204	62
	Recife (PE)	1990	15	84	460	58
	Maceió (AL)	501	9	5	98	34
	Aracaju (SE)	526	11	58	224	39
Southeast	Belo Horizonte (MG)	3584	23	76	652	104
	Vitória (ES)	780	28	168	1081	116
	Rio de Janeiro (RJ)	13249	32	47	372	34
	São Paulo (SP)	51311	70	46	306	108
Southern	Florianópolis (SC)	5843	176	281	1990	624
	Curitiba (PR)	5198	41	72	505	183
	Porto Alegre (RS)	4471	46	167	1091	498
Midwestern	Goiânia (GO)	3836	48	61	408	60
	Cuiabá (MT)	729	10	110	354	142

	Campo Grande (MS)	2160	50	48	391	30
	Brasília (DF)	3546	13	12	96	17

*PrEP users with at least 1 drug refill preceded by a HIV testing in each year assessed during the study period.
