# Supplementary material for: HIV pre-exposure prophylaxis and incidence of sexually transmitted infections in Brazil, 2018 to 2022: An ecological study of PrEP administration, syphilis, and socioeconomic indicators
Source: PLoS Negl Trop Dis. 2023 Aug 11;17(8):e0011548. doi: 10.1371/journal.pntd.0011548 (PMC10446216; doi:10.1371/journal.pntd.0011548)
Supplement: S2 Table — (PDF) [file pntd.0011548.s002.pdf]

Supporting information

HIV pre-exposure prophylaxis and incidence of sexually transmitted infections in Brazil, 2018 to 2022: an ecological study of PrEP administration, syphilis, and socioeconomic indicators

Paula Knoch Mendonça Gil, Danilo dos Santos Conrado, Ana Isabel do Nascimento, Micael Viana de Azevedo, João Cesar Pereira da Cunha, Gabriel Serrano Ramires Koch, Camila Guadeluppe Maciel, Alisson André Ribeiro, Antonio Conceição Paranhos Filho, Márcio José de Medeiros, Cláudia Du Bocage Santos-Pinto, Everton Falcão de Oliveira

S2 Table. PrEP dispensing and STI occurrence per 1000,000 inhabitants by Brazilian state capitals by year, 2018–2022

Brazilian region	State capital (State)	Year	PrEP administration*	PrEP administrations per 100,000 inhab.	HIV/AIDS	Syphilis	Viral hepatitis
Northern	Manaus (AM)	2018	352	24	56	229	54
		2019	660	43	66	282	61
		2020	652	42	54	222	32
		2021	991	62	33	119	22
		2022	1305	82	26	126	0

Rio Branco (AC)	2018	1	0	26	278	118
	2019	1	0	21	212	105
	2020	28	10	12	178	33
	2021	110	38	6	158	52
	2022	151	52	13	95	0
Porto Velho (RO)	2018	3	1	65	278	157
	2019	16	4	61	252	133
	2020	45	12	50	262	67
	2021	98	25	23	157	59
	2022	154	39	21	94	0
Boa Vista (RR)	2018	19	7	63	222	57
	2019	56	20	60	234	66
	2020	71	24	32	177	43
	2021	157	51	14	79	39
	2022	234	75	20	90	0
Macapá (AP)	2018	15	5	45	139	17
	2019	32	9	39	164	10
	2020	31	9	31	114	4
	2021	78	22	16	89	4
	2022	187	52	16	108	0

	Belém (PA)	2018	27	2	155	319	50
		2019	175	16	161	327	57
		2020	318	28	111	297	27
		2021	422	37	55	183	30
		2022	618	54	24	38	0
	Palmas (TO)	2018	15	7	79	673	70
		2019	48	23	59	637	51
		2020	41	19	51	487	24
		2021	96	44	26	289	25
		2022	143	65	10	127	0
Northeast	São Luís (MA)	2018	17	2	129	335	48
		2019	46	6	118	305	47
		2020	77	9	82	222	18
		2021	218	26	32	132	25
		2022	315	38	17	49	0
	Teresina (PI)	2018	6	1	45	182	17
		2019	93	15	51	220	18
		2020	120	19	39	133	8
		2021	192	29	16	48	6
		2022	311	48	12	25	0

Natal (RN)	2018	87	13	84	284	24
	2019	171	25	69	303	21
	2020	192	28	53	267	12
	2021	398	57	21	172	10
	2022	642	93	17	93	0
Fortaleza (CE)	2018	217	11	52	186	18
	2019	451	22	52	193	15
	2020	678	33	39	172	10
	2021	948	46	14	83	9
	2022	1538	755	8	38	0
João Pessoa (PB)	2018	85	14	71	282	33
	2019	159	26	75	308	32
	2020	161	26	59	194	17
	2021	268	43	24	104	15
	2022	413	66	9	52	0
Salvador (BA)	2018	228	10	67	456	55
	2019	443	20	67	305	59
	2020	534	24	55	204	31
	2021	747	33	23	149	25
	2022	1104	49	13	67	0

	Recife (PE)	2018	136	11	116	645	34
		2019	261	21	113	668	45
		2020	256	20	84	460	29
		2021	413	32	38	272	24
		2022	924	71	13	77	0
	Maceió (AL)	2018	3	0	10	147	31
		2019	51	7	8	116	32
		2020	95	13	5	98	17
		2021	137	18	3	47	14
		2022	215	28	12	26	0
	Aracaju (SE)	2018	4	1	62	224	40
		2019	54	11	57	199	37
		2020	72	14	58	224	20
		2021	142	28	26	182	23
		2022	254	50	15	74	0
Southeast	Belo Horizonte (MG)	2018	185	9	100	770	103
		2019	507	25	96	787	94
		2020	584	29	76	652	52
		2021	869	43	35	327	38

		2022	1439	71	9	101	0
	Vitória (ES)	2018	33	12	184	1769	164
		2019	98	35	199	1814	132
		2020	102	36	168	1081	58
		2021	199	69	65	642	83
		2022	348	121	11	198	0
	Rio de Janeiro (RJ)	2018	1142	22	63	373	38
		2019	1875	36	60	399	31
		2020	2137	40	47	372	17
		2021	2501	47	21	151	15
		2022	5594	105	12	84	0
	São Paulo (SP)	2018	2723	29	58	414	109
		2019	5269	56	53	384	91
		2020	8644	91	46	306	54
		2021	14594	153	20	161	44
		2022	20081	210	8	97	0
Southern	Florianópolis (SC)	2018	315	80	388	2849	517
		2019	720	180	364	2459	490
		2020	897	220	281	1990	312
		2021	1589	384	135	1166	265
		2022	2322	561	14	22	0

	Curitiba (PR)	2018	325	22	101	672	170
		2019	881	59	97	706	161
		2020	803	53	72	505	92
		2021	1317	86	32	219	67
		2022	1872	122	9	91	0
	Porto Alegre (RS)	2018	495	43	215	1327	436
		2019	716	61	219	1319	414
		2020	686	58	167	1091	249
		2021	911	77	71	508	207
		2022	1663	141	18	48	0
Midwestern	Goiânia (GO)	2018	70	6	70	457	49
		2019	266	23	74	492	49
		2020	739	63	61	408	30
		2021	1140	96	28	221	52
		2022	1621	137	11	95	0
	Cuiabá (MT)	2018	6	1	124	420	117
		2019	26	6	143	446	128
		2020	63	14	110	354	71
		2021	202	43	39	106	54
		2022	432	93	11	15	0

Campo Grande (MS)	2018	4	1	72	697	40
	2019	173	26	76	522	32
	2020	454	67	48	391	15
	2021	634	92	29	162	11
	2022	895	131	12	22	0
Brasília (DF)	2018	264	12	14	78	10
	2019	489	22	15	91	10
	2020	406	18	12	96	9
	2021	745	32	6	45	7
	2022	1642	70	6	40	0

*PrEP users with at least 1 drug refill preceded by a HIV testing in each year assessed during the study period.
