# Supplementary material for: HIV pre-exposure prophylaxis and incidence of sexually transmitted infections in Brazil, 2018 to 2022: An ecological study of PrEP administration, syphilis, and socioeconomic indicators
Source: PLoS Negl Trop Dis. 2023 Aug 11;17(8):e0011548. doi: 10.1371/journal.pntd.0011548 (PMC10446216; doi:10.1371/journal.pntd.0011548)
Supplement: S3 Table — (PDF) [file pntd.0011548.s003.pdf]

Supporting information

HIV pre-exposure prophylaxis and incidence of sexually transmitted infections in Brazil, 2018 to 2022: an ecological study of PrEP administration, syphilis, and socioeconomic indicators

Paula Knoch Mendonça Gil, Danilo dos Santos Conrado, Ana Isabel do Nascimento, Micael Viana de Azevedo, João Cesar Pereira da Cunha, Gabriel Serrano Ramires Koch, Camila Guadeluppe Maciel, Alisson André Ribeiro, Antonio Conceição Paranhos Filho, Márcio José de Medeiros, Cláudia Du Bocage Santos-Pinto, Everton Falcão de Oliveira

S3 Table. Socioeconomic indicators of Brazilian state capitals, Brazil, 2010

State capitals (State)	Illiteracy rate (%)	Gini coefficient	Per capita income (BRL)	Garbage collection (%)	Sanitary facilities (%)	Water supply (%)
Manaus (AM)	3.9	0.6334	738.42	94.04	39.99	75.71
Rio Branco (AC)	8.9	0.6121	703.82	86.28	43.47	52.31
Porto Velho (RO)	5.2	0.5745	881.25	84.36	09.28	36.97
Boa Vista (RR)	5.7	0.5936	752.13	93.06	16.92	95.08
Macapá (AP)	6	0.6037	690.04	90.50	08.36	55.36
Belém (PA)	3.3	0.6284	812.43	90.32	36.66	76.00

Palmas (TO)	3.7	0.5914	1060.21	9276	39.78	94.06
São Luís (MA)	4.6	0.6266	770.52	85.54	44.73	75.85
Teresina (PI)	8.8	0.6171	739.85	89.55	17.41	92.52
Natal (RN)	7.9	0.6217	921.29	94.98	30.46	97.66
Fortaleza (CE)	6.8	0.6267	812.61	92.50	57.91	92.48
João Pessoa (PB)	7.7	0.6287	934.74	95.33	54.65	95.40
Salvador (BA)	3.9	0.6449	935.66	59.99	89.58	98.06
Recife (PE)	6.9	0.6894	1109.01	93.75	52.58	86.58
Maceió (AL)	11.3	0.6378	773.26	86.03	28.88	73.69
Aracaju (SE)	6.6	0.6341	1022.07	92.05	70.76	96.96
Goiânia (GO)	3.1	0.5908	1305.36	93.47	67.06	92.03
Cuiabá (MT)	4.5	0.4691	1124.88	91.30	56.26	93.27
Campo Grande (MS)	3.8	0.5720	1071.17	96.87	41.93	89.48
Brasília (DF)	3.6	0.6370	1665.42	83.33	78.01	93.82
Belo Horizonte (MG)	2.8	0.6106	1455.52	96.89	94.92	98.96
Vitória (ES)	2.5	0.6124	1820.97	95.04	95.90	98.56
Rio de Janeiro (RJ)	2.7	0.6391	1421.76	83.54	89.36	97.43
São Paulo (SP)	3.1	0.6453	1416.13	94.01	90.09	98.27
Florianópolis (SC)	1.9	0.5474	1770.29	91.91	50.18	92.30
Curitiba (PR)	2.1	0.5652	1536.39	96.19	90.98	98.48
Porto Alegre (RS)	2.2	0.6144	1722.37	95.27	82.95	98.21
