# Supplementary figures and images for: HIV pre-exposure prophylaxis and incidence of sexually transmitted infections in Brazil, 2018 to 2022: An ecological study of PrEP administration, syphilis, and socioeconomic indicators
Source: PLoS Negl Trop Dis. 2023 Aug 11;17(8):e0011548. doi: 10.1371/journal.pntd.0011548 (PMC10446216; doi:10.1371/journal.pntd.0011548)

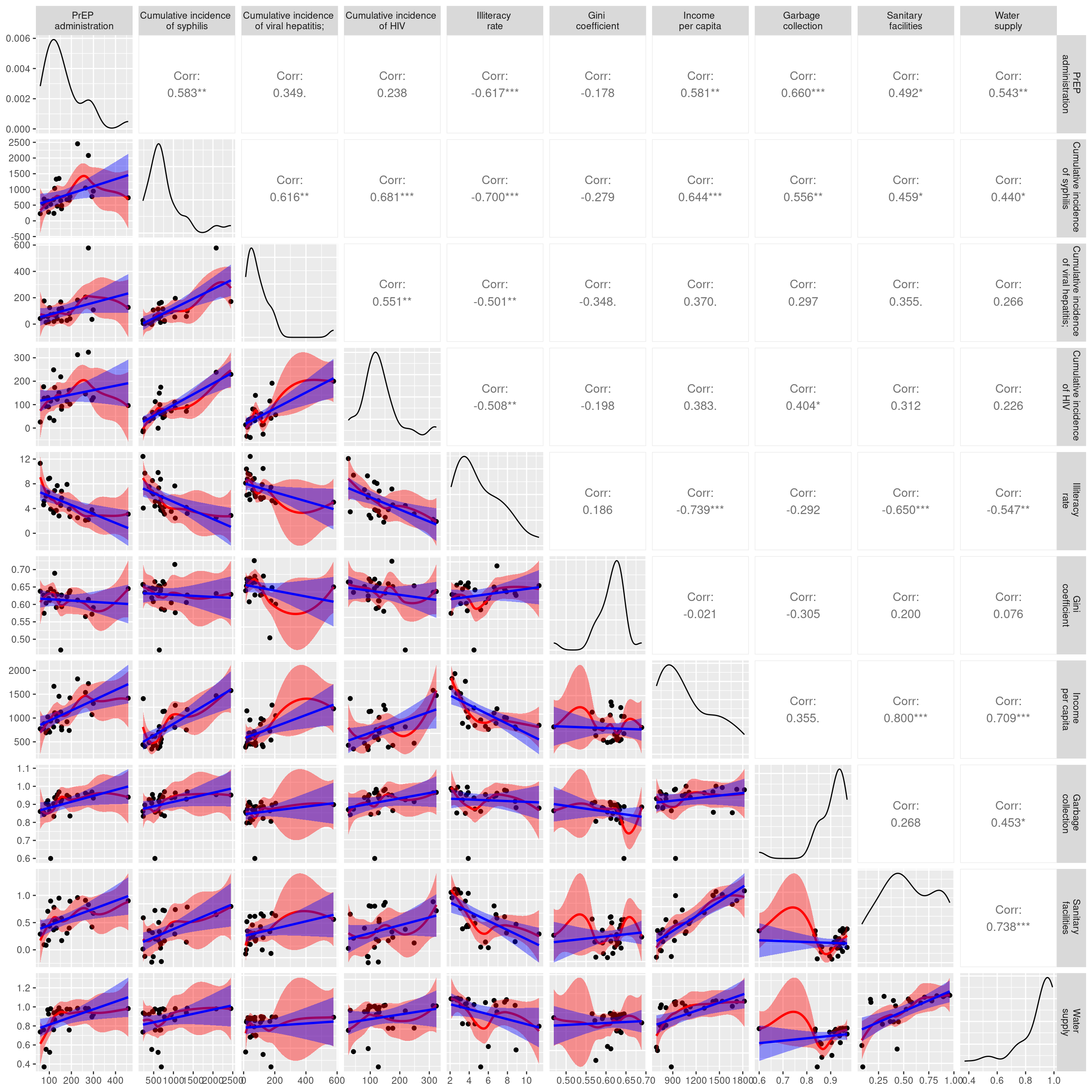

Supplement: S1 Fig — ***p<0.001; **p<0.05; *p = 0.10 (TIFF) [file pntd.0011548.s004.tiff]
